# Perspectives on the Combined Effects of *Ocimum basilicum* and *Trifolium pratense* Extracts in Terms of Phytochemical Profile and Pharmacological Effects

**DOI:** 10.3390/plants10071390

**Published:** 2021-07-07

**Authors:** Andreea-Ina Antonescu (Mintas), Florina Miere (Groza), Luminita Fritea, Mariana Ganea, Mihaela Zdrinca, Luciana Dobjanschi, Angela Antonescu, Simona Ioana Vicas, Florin Bodog, Rakesh K. Sindhu, Simona Cavalu

**Affiliations:** 1 Faculty of Medicine and Pharmacy, Doctoral School of Biomedical Science, University of Oradea, 10 P-ta 1 December Street, 410073 Oradea, Romania; antonescu.ina@gmail.com; 2 Faculty of Medicine and Pharmacy, University of Oradea, 10 P-ta 1 December Street, 410073 Oradea, Romania; florinamiere@uoradea.ro (F.M.); mganea@uoradea.ro (M.G.); mzdrinca@uoradea.ro (M.Z.); dobjanschil@uoradea.ro (L.D.); fbodog@uoradea.ro (F.B.); scavalu@uoradea.ro (S.C.); 3 Faculty of Environmental Protection, University of Oradea, 26 Gen. Magheru Street, 410048 Oradea, Romania; svicas@uoradea.ro; 4 Chitkara College of Pharmacy, Chitkara University, Punjab 140401, India; rakesh.sindhu@chitkara.edu.in

**Keywords:** *Ocimum basilicum* sp., *Trifolium pratense* sp., phytochemistry, antioxidant capacity, antibacterial activity, wound healing, anti-inflammatory

## Abstract

Nowadays, the tendency in pharmaceutical and food industries is to replace synthetic antioxidants with the natural ones. For this reason, there is a growing interest in analyzing natural, healthy and non-toxic additives as potential antioxidants. Some plants, which contain high levels of phenolic compounds, present an increasing interest for medicine due to their ability to scavenge free radicals, along with other pharmacological activities, such as antibacterial activity, wound healing and anti-inflammatory effect, to mention only a few. The aim of this review is to explore the therapeutic potential of *Ocimum basilicum* and *Trifolium pratense* in relation with their phytochemical profile and to highlight the pharmacological activity of aqueous or ethanol extracts. Special attention was devoted to the dermal pathology and wound healing effects, in the context of multiple skin conditions such as acne, eczema boils, psoriasis and rashes. Additionally, both extracts (*Trifolium* sp. and *Ocimum* sp.) are characterized by high content of antioxidant compounds, which are responsible for the radiance and resistance of the skin and slowing down of the aging process by maintaining estrogen levels. Moreover, the potential combined effect of the mixed extract is pointed out in terms of future applications for wound healing, based on some preliminary results obtained from a “scratch tests” assay performed with respect to human dermal fibroblasts.

## 1. Introduction

Plants have been sources of minerals, vitamins and bioactive compounds since ancient times that are used in traditional medicine to treat various diseases. The biologically active compounds from the plants have been the natural elements from which allopathic treatments and synthetic medicinal substances have developed over time and they are currently used successfully in the treatment of multiple diseases [1]. However, the World Health Organization has estimated that 80% of the population prefer plants-based treatments to treat respiratory and dermatological diseases, cancer, oxidative stress, diabetes, etc [2]. Plants are rich sources of biochemical compounds such as phenols, fatty acids, saponins, essential oils or alkaloids which have proven therapeutic properties but are less studied and valued [3].

*Ocimum* and *Trifolium* species are plants that have also not been valued in the past, but there is an increase interest in these two plants species in recent years [4,5]. *Ocimum* species is characterized by an abundance of compounds such as phenolic acids, but also volatile oils, and *Trifolium* species have been shown to be rich in biologically active compounds such as isoflavones [6,7].

*Ocimum sp.* belongs to the *Lamiaceae* family, one of the largest plant families comprising about 220 genera and almost 4000 species. The most important representatives of *Ocimum* sp. are: *Ocimum basilicum, Ocimum sanctum, Ocimum gratissimum, Ocimum canum, Ocimum kilimandscharicum, Ocimum americanum* and *Ocimumm icranthum* [8,9,10].

*Ocimum basilicum* L., called sweet basil, is botanically described as a branched plant that grows between 0.3 and 1.3 m height, with light green silky leaves in opposite directions and containing many oily glands that store essentials oils [11]. The basil flowers are colored from white to purple and arranged in a terminal spike [11].

The members of genus *Ocimum* are very important for their therapeutic potentials being used against abdominal cramps, gastroenteritis, dysentery, and diarrhea. The leaves extract was used in the treatment of wounds, acne, and vitiligo. Additionally, basil has been utilized traditionally for curing health issues such as: anxiousness, stings, strong aching, gripe, pyrexia, infective diseases, headaches, coughs, constipation, warts, worms and kidney malfunction [12,13,14]. It was also used as a deodorant, being considered to be an aphrodisiac [15].

The genus *Trifolium* is one of the most important genus in the *Leguminosae* family comprising over 240 species, being remarkable for its therapeutic effects such as: expectorant, analgesic, antioxidant and anti-inflammatory [16]. Some representatives species are: *Trifolium repens* (white clover), *Trifolium pratense* (red clover), *Trifolium fragiferum* and *Trifolium hybridum*. The Mediterranean region is very rich in *Trifolium* species, represented by 103 species [17].

The purpose of this paper is to concentrate the information from the literature about the two plants mentioned before (*Trifolium* sp. and *Ocimum* sp.). The plants characteristics are described in terms of chemical composition and therapeutic activity of different types of extracts.

This review also considers the combination of the two species, based on the common bioactive compounds identified in their composition, along with their similar therapeutic activities. One of these similar biological activities is the healing of wounds. Some preliminary results confirm the high therapeutic potential combining the extracts from both plants, working synergistically with great benefits.

## 2. Common Therapeutic Activities of Ocimum and Trifolium Species

**Figure 1:**
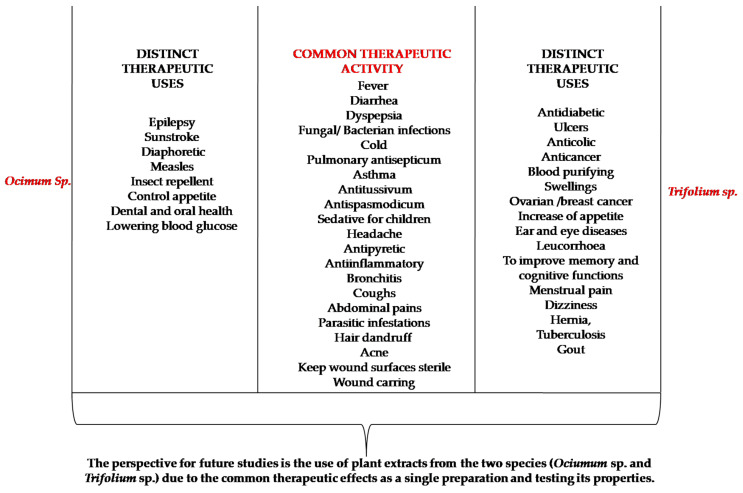
Common and distinct therapeutic effects of *Ocimum* and *Trifolium* species.

*Ocimum* species have been shown to have multiple therapeutic effects in the area of respiratory diseases, wound treatment, bacterial or fungal infections, headaches, and gastrointestinal disorders. Meanwhile, *Trifolium* species have been demonstrated to present multiple therapeutic effects in the field of respiratory diseases, wound treatment, bacterial or fungal infections, gastrointestinal disorders, menstrual pain, anticancer, antidiabetic, tuberculosis, to mention only a few [5,7,18]. So, there are some common therapeutic activities, but also some distinct therapeutic effects of the two species as presented in Figure 1.

However, in skin wounds, including acute wounds and chronic wounds, the application of different formulations based on plants extracts for wound healing involve a dynamic and complex process for recovering tissue integrity and homeostasis: inflammation, reepithelization, granulated tissue formation, neovascularization, wound contraction and remodeling of the extracellular matrix. Hence, the potential of *Ocimum* and *Trifolium* species to enhance the healing process is far from being completely explored and new therapeutic options with fewer adverse effects, low cost and reduced healing time are still required for clinical or alternative treatments.

## 3. Phytochemistry

### 3.1. Phytochemical Profile of Ocimum Species

*Ocimum* is noted for its pungency and flavor apart from its aroma. The aroma in this genus is due to the essential oil, its contents ranging from 0.3% to 3.6% dry weight. The minor components in this genus, most of which are sesquiterpenes, are found to vary amongst species [11]. The chemical composition of *Ocimum basilicum* essential oil has been studied in various parts of the world [19]. Many authors isolated the essential oil from *Ocimum basilicum* reporting various volatile constituents. The main constituents are: linalool, 1,8-cineol, eugenol, methyl cinnamate, camphor, methyl eugenol, methyl chavicol, β-elemene, β-ocimene, camphene, carvacrol, α-bergamotene, α-cadinol and geranial [4,11,19,20].

Basil has been classified according to different geographical origins. There are many chemotypes such as: the European chemotype from Italy, France, Bulgaria, Romania, Egypt, and South Africa, having linalool and methyl chavicol as main components; the tropical chemotype from India, Pakistan and Guatemala, being rich in methyl cinnamate; and the Reunion chemotype from Thailand, Madagascar and Vietnam, being characterized by high concentration of methyl chavicol. There is also a eugenol-rich chemotype from North Africa and Russia [11,13]. Other chemotypes were reported in recent studies such as ß-caryophyllene in *O. sanctum* and *Ocimum micranthum,* citral in *Ocimum citriodorium* and *Ocimum canum*, ethyl cinnamate in *Ocimum gratissimum*, 1,8-cineole in *Ocimum micranthum*, thymol in *Ocimum gratissimum*, *p*-cymene in *Ocimum gratissimum*, geranyl acetate in *Ocimum minimum*, and camphor in *Ocimum canum* [9,21,22].

Recently, the hydroalcoholic extract of *Ocimum basilicum* harvested from Romania has been characterized in terms of total polyphenolic compounds, identifying cinnamic acid, caffeic acid, ferulic acid, syringic acid, catechin, rutin and chlorogenic acid [23]. The main antioxidant compounds in basil extracts are chlorogenic, p-hydroxybenzoic, caffeic, vanillic and rosmarinic acids, as well as apigenin, quercetin and rutin [20]. Ferulic acid were also identified by other authors [24]. These compounds were also identified in *Ocimum basilicum* by Dhama et al., pointing out its huge therapeutic potential [25].

### 3.2. Phytochemical Profile of Trifolium Species

**Figure 2:**
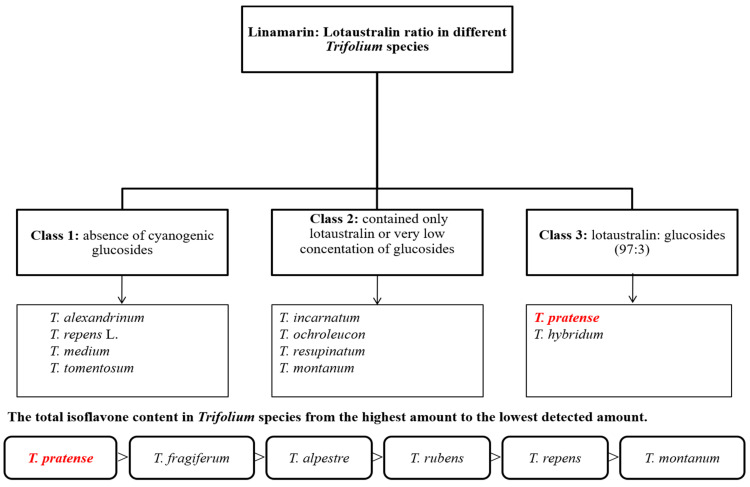
Grouping the different species of *Trifolium* according to the amount of cyanogenic glycosides and isoflavones and identifying the species with rich chemical composition.

Until 2012, studies on *Trifolium* plants phytochemistry were mostly focused on *Trifolium pratense* or *Trifolium repens* [26]. Recent years have provided a noticeable growth in research on different clovers as a source of bioactive substances. Muzashvili et al. [27] have studied the composition of 88 species of *Trifolium* highlighting compounds such as linamarin and lotaustraline, which are part of the class of cyanogenic glycosides [27]. It has been shown by the phytochemical characterization that the ratio between the two glycosides is different from one species to another, depending on the harvest period. Plants harvested in the early period of the flowering phase presented the highest content of glycosides. Thus, *Trifolium* species can be grouped into three major classes, taking into account the amount of cyanogenic glycosides and their ratio in the extract of the aerial part of the plant [27,28,29] (Figure 2).

Several species of clovers were also a subject of previous study [30] focused on the distribution of three isoflavones (formononetin, daidzein and genistein) in *Trifolium* species. Total isoflavones were quantified from clover leaves, stems and flowers.

Figure 2 shown the *Trifolium* species that contain amounts of isoflavones in descending order. Among the *Trifolium* species the richest in isoflavones is *T. pratense* [27,31,32].

*Trifolium pratense* is a rich source of isoflavonoids (biochanin A, daidzein, formononetin, afrormosin, orobol, genistein, pratensein, trifoside) and flavonoids (quercetin and kaempferol) [7,30,31,33]. Other constituents include medicagol, coumestrol, coumarin. The components found abundantly in the roots are biochanin A, afrormosin, daidzein, genistein, methyl orobol, irilin and irilone, meanwhile the components from the leaves are formononetin, biochanin A, soyasaponins, clovamides and flavonoids [7,27,31,33].

Antonescu et al. [23] have identified in the *T. pratense* extract the following phenolic compounds: cinnamic acid, caffeic acid, ferulic acid, syringic acid, catechin, rutin and chlorogenic acid [23].

## 4. Pharmacological Activities

**Figure 3:**
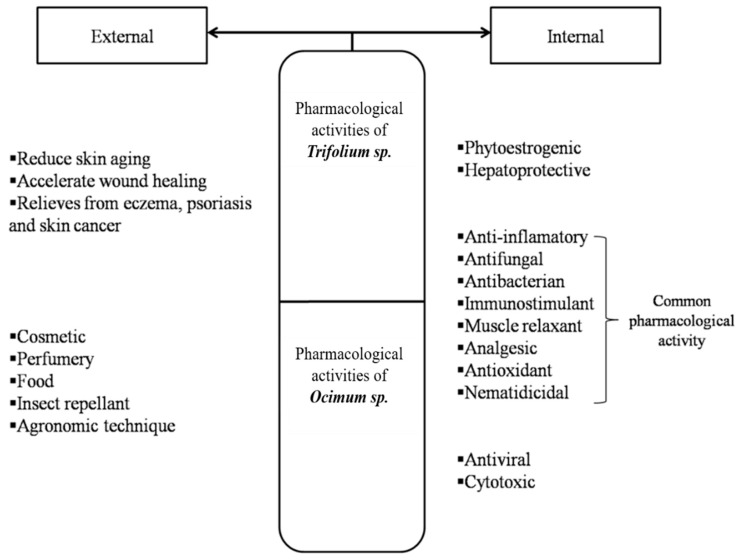
Pharmacological activities of extracts from *Trifolium* and *Ocimum* species after internal and external administration.

Due to some common compounds the extracts from the plants *T. pratense* and *O. basilicum* have been shown to have similar therapeutic effects [34,35,36]. The biological activities specific to the *T. Pratense* and *O. basilicum*, but also its common biological effects are presented in Figure 3 [11,28].

The chemical structures of the major compounds of the *Ocimum* and *Trifolium* species and the specific therapeutic activities are presented in Table S1 (for *Ocimum* sp.) and Table S2 (for *Trifollium* sp.).

### 4.1. Antioxidant Capacity of Ocimum and Trifolium Species

Nowadays, the tendency in pharmaceutical and food industries is to replace synthetic antioxidants with the natural ones. For these reasons there is a growing interest in analyzing natural, healthy and non-toxic additives as potential antioxidants [28,37,38]. Some plants, which contain high level of phenolic compounds, present an increasing interest in medicine due to their ability to scavenge free radicals [39,40,41]. It has been noticed a direct correlation between the antioxidant activity and the phenols concentration. Phenols are very important bioactive compounds because they are acting as scavengers of intermediate peroxyl and alkoxyl radicals, and chelating agents for metal ions which are of major importance for the initiation stage of radical reactions [42].

*Ocimum and Trifolium* sp. contain many antioxidant compounds which contribute to their intense antiradical activity [43,44] and could have potential human health benefits [21]. Due to the strong antioxidant capacity, basil acts as a protector to prevent heart diseases, reduce inflammation, lower the incidence of cancers and diabetes [40]. A very strong correlation was demonstrated between the some phenolic compounds and antioxidant capacity of medicinal plants [45,46]. *Ocimum basilicum* extracts possess a higher total phenolic acid content and greater antioxidant activity.

The aqueous extract of basil is a superoxide and hydroxyl radical scavenger [43,47]. The antioxidant capacity of this extract has been attributed to its polar phenolic compounds. The total phenolic content of water and ethanol extracts of basil was reported to be similar in the linoleic acid peroxidation (94.8% and 97.5%) [48]. Hinneburg et al. [49] reported that hydrodistilled extracts from basil had the highest antioxidant capacity in comparison with several herbs like laurel, parsley, juniper, aniseed, fennel, cumin, cardamom, and ginger, but not the greatest iron chelation ability [49]. Rosmarinic acid has been identified as the primary phenolic compound in basil leaves and stems [36]. Chicoric acid has also been identified in substantial quantities [36].

Extracts of *Trifolium pratense* are becoming increasingly popular, primarily for the treatment of menopausal symptoms [47,50,51]. Furthermore, phytoestrogens present in *T. pratense* are also effective antioxidants and may have tyrosine kinase inhibitory activity. The antioxidant properties of genistein and other phytoestrogens have been demonstrated in several models such as protection from phorbol ester-induced singlet oxygen or peroxide formation and particularly from UV-radiation-induced oxidative damage to DNA in vitro [52,53]. Dietary genistein has been shown during the in vivo experiments in mice to stimulate the endogenous antioxidants, SOD (superoxide dismutase), GSHP (glutathione peroxidase) and glutathione S-transferase, with the effects found mainly in small intestine and the skin [54,55,56]. Sanja Vlaisavljevic et al. [57] studied the antioxidant capacity of the extracts by using tests that are based on electron transfer (neutralization of DPPH radical), neutralization of free radical species (capacity of scavenging O_2_, OH and NO radicals) and the potential to inhibit lipid peroxidation [36,57,58].

The highest concentration of phenolic and flavonoid substances was found in methanol extract isolated from plants cultivated in vivo condition which displayed the highest reducing ABTS radical scavenging and chelating abilities [23]. However, the most effective scavenger of DPPH radical, superoxide anion and hydrogen peroxide, was the chloroform fraction of red clover grown in vivo. The current state of understanding the antioxidant actions of clover species is still mostly based on in vitro experimental systems [58,59].

Besides phenols, some volatile compounds were demonstrated to possess antioxidant activity. Araujo Couto et al. [6] indicated that the major essential oil compound (eugenol) has been corelated with high antioxidant capacity. Necar and Tansi have highlighted linalool as a major compound in Greek basil [60]. Linalool, epi-*α*-cadinol, and *α*-bergamotene (7.4% to 9.2%) and *γ*-cadinene have been identified as the most common compounds in basil essential oil. Basil essential oil strongly inhibits lipid peroxidation whether induced by Fe^2+^/ascorbate or by Fe^2+^/H_2_O_2_.

Al-Maskria et al. have demonstrated that the essential oil content and antioxidant capacity varied in function of the season when it was harvested (the antioxidant capacity was the highest in spring) [61]. Politeo et al. investigated antioxidant activity measured by DPPH revealing that free volatile compounds (eugenol, chavicol, linalool and α-terpineol) possess good antioxidant properties comparable with that of the essential oil and well-known synthetic antioxidant butylated hydroxytoluene (BHT), but less than pure eugenol [37].

The recent literature evidenced antioxidant properties of several *Trifolium* species (*Trifolium angustifolium, Trifolium balansae, Trifolium stellatum, Trifolium nigrescens, Trifolium constantinopolitanum, Trifolium pallidum and Trifolium resupinatum*), but only the antioxidant action of Trifolium pratense was examined in vivo [26,29]. Recently, antioxidant action has become one of the most studied properties of clovers (after the estrogenic effect) [28,29]. The antioxidant activity of glycosidically bound volatile compounds in clover essential oil has been reported to be significantly greater than that of the volatile aglycones [62]. The glycosides can undergo enzymatic hydrolysis releasing their aglycones, therefore, they may be considered as potential antioxidant precursors [63,64].

### 4.2. Antimicrobial, Antiviral and Antifungal Activity of Ocimum and Trifolium Species

Essential oil present in most of the *Ocimum* species is responsible for its antifungal, antibacterial and antiviral properties. The essential oils of various *Ocimum* species have been shown, in vitro, to have antibacterial activity against *Staphylococcus aureus, Salmonella enteritidis, Escherichia coli, Proteus vulgaris, Bacillus subtilis, Salmonella typhi, Shigella sonnei, Shigella boydii, Pseudomonas aeruginosa* and *Salmonella paratyphi* [8,57,58].

The *Ocimum basilicum* oil was tested also against pathogenic fungi such as: *Aspergillus niger, Aspergillus fumigatus, Penicillium italicum* and *Rhizopus stolonifera* by using a disc diffusion method, and by determination of minimum inhibitory concentration. Surprisingly, high antifungal effect were found highlighting the potential of *Ocimum* species as a preservative in food and medical industries [61].

Studies have shown *Ocimum basilicum* act as a strong antiviral agent against DNA viruses (herpes simplex viruses, adenoviruses and hepatitis B virus) and RNA viruses (coxsackievirus and enterovirus). *Ocimum tenuiflorum* has been also reported to have antiviral activity against bovine herpesvirus 1 [65,66,67]. Another study investigated the influence of honey and some surfactants (cationic, anionic) on the antibacterial activity of *Ocimum gratissimum* essential oil pointing out that honey was more efficient than a macrogol bend due to which it could be suitable for the infected wounds treatment [63].

Zahran et al., also emphasized the complex composition of *Ocimum* species extract, which determines its strongly anti-inflammatory, antibacterial and antiviral activity. The authors also indicated a classification of the most powerful therapeutic species of *Ociumum,* which are: *Ocimum basilicum*, *Ocimum sanctum* and *Ociumum gratissiumum* [68].

The literature about the antimicrobial activity of clover species contains the evaluation of the efficiency of plant extracts from *Trifolium* species [69,70,71]. Testing of the antimicrobial and antifungal activity of the extracts of these species was performed on gram-positive bacteria (*Streptococcus pyogenes* and *Staphylococcus aureus*), gram-negative bacteria (*Pseudomonas aeruginosa*, *Escherichia coli*) and fungi (*Candida albicans*). According to the literature, the extraction solvent of the active principles has a major importance on the antibacterial and antifungal activity of the *Trifolium* species [72]. Studies on the antimicrobial properties of *Trifolium pratense* included a comparison of the actions of different extracts (using solvents such as ethanol, methanol, water, ether), all pathogens examined were inhibited by the extract made in methanol, which was declared to have the highest antibacterial and antifungal activity [46,69].

### 4.3. Anti-Inflammatory Effect of Ocimum Species

Up to date, basil extract has been experimented on rats to reduce acute inflammation. Basil alcohol extract has been shown to have a slight effect on nitrogen oxide synthesis, but it has reduced the number of leukocytes and monocytes, as well as significantly activated circulating phagocytes. The highlighting of the anti-inflammatory effect of basil species extract was compared with diclofenac, the extract presenting less intense activity compared to it [24].

The oils of different species of *Ocimum* (*Ocimum* *sanctum*, *Ocimum* *basilicum*, *Ocimum* *americanum*), showed a different response against edema. *Ocimum* *bazilicum* oils possess the highest percentages of linolenic acid (21.0%) and provided maximum inhibition of edema (72.42%) [73]. The oil can inhibit increased vascular permeability and leukocyte migration, as evidenced by the inflammatory stimulus [73,74].

The anti-inflammatory effect of *Ocimum* species has also been studied in the ear [75]. According to this study, it was found that when applying 50 μg extract in the ear, the inflammation and edema at this level is significantly reduced by 80% due to its local anti-inflammatory effect. The effects were comparable to 100 μg hydrocortisone as a control, showing an inhibition of 54.8%.

In another study was investigated the immunomodulatory effect of *Ocimum* *sanctum* seed oil on immunological parameters in both stress-free and stressed animals and assessed that this oil appears to modulate both the humoral immune reaction, as well as the immediate one and these immunomodulatory effects can be mediated by the GABA-ergic pathway [76].

### 4.4. Estrogenic and Anticancer Activity of Trifolium Species

#### 4.4.1. Estrogenic Action

Lately, much emphasis has been placed on the estrogenic effects of different types of isoflavonic compounds of *Trifolium* species. In general, research on the estrogenic properties of *Trifolium pratense* has recently been extended by more detailed examinations of the pharmacological role of individual isoflavones, which is a new issue in investigations of the phytoestrogenic action of this plant. It has been shown in in vivo studies that daidzein and genistein are the main isoflavonoid compounds that are present in blood plasma after administration of *Trifolium pratense* extracts. However, recent studies have shown that the bioavailability of several isoflavones present in *Trifolium pratense* is increased, these being irilone, prunetine and pseudobaptigenin [77].

In addition, it is suggested that after consuming a red clover dietary supplement, irilone may be the second most abundant isoflavone in human plasma, along with daidzein. The estrogenic action of irilone and daidzein was evaluated compared to different estrogens [78]. Irilone has been shown to significantly increase alkaline phosphatase activity, as well as induce mRNA for this enzyme, progesterone receptors and androgen receptor mRNA levels. Experiments performed on cells showed that irilone significantly induced their proliferation [78]. The studies conducted by Spagnuolo P et al. showed that the estrogenic effect of *Trifolium* species extracts is dependent and increasing with the administered dose [79].

#### 4.4.2. Anticancer Activity

The anticancer activity of *Trifolium pratense* is given by the extract’s ability to determine cell regeneration [59]. The active ingredients in *Trifolium pratense* have been shown to be used as an adjunct in the treatment of cancer in combination with other medicinal plants in the form of an internal infusion or tincture [59,80]. The 95% ethyl alcohol extract of *Trifolium pratense* significantly inhibited the metabolism of cancer cells and decreased the level of binding of benzopyrene to DNA by 30 to 40% [80]. Biochanin A has also been shown to be an isolated isoflavone and identified as a major active compound of *Trifolium pratense* extract. The ability of this isoflavone to inhibit carcinogen activation in culture cells suggests that in vivo studies of this compound as a potential chemopreventive agent are warranted [42,80]. Up to date, no anticancer activity has been found on breast cancer (including estrogenic activity) and hepatocellular carcinoma, but promising anticancer activity of aqueous *Trifolium pratense* extract on gastric or colon cancer has been demonstrated [81].

### 4.5. Dermal Pathology and Wound Healing Effects of Ocimum and Trifolium Species

Recent literature has shown the beneficial effects of *Trifolium* and *Ocimum* species extracts on skin health. Various concentrations of *Ocimum gratissimum* oil were tested compared to benzoyl peroxide 10% and a placebo over a four-week period to reduce the acne lesions in a predominantly student population. *Ocimum gratissimum* oil in different concentrations of 0.5%, 1%, 2% and 5% *v/v* were incorporated in various topical formulations. This study showed that preparations containing 2% and 5% *Ocimum* oil in alcohol and 5% in ketomacrogol were significantly more active than benzoyl peroxide [82].

Another clinical study was performed using a combination of *Ocimum gratissimum* and Aloe vera gel. Aloe vera gel has been found to improve the anti-acne properties of *Ocimum* oil. The oil or its combination with Aloe vera gel has been shown to be more effective than 1% clindamycin in the treatment of acne vulgaris [83]. In another study by Pansanga et al. it was found that a microemulsion of *Ocimum* species 3% should be safe and well tolerated on human skin [84].

Renda et al. described the in vivo wound healing effects of aqueous-methanolic extracts of 13 species of Trifolium [85]. The effects of Trifolium extracts in animals were compared with the reference medicine Madecassol, whose activity was assumed to be 100%. The most effective wound healing properties were found for *Trifolium canescens*, the second was Trifolium pratense extract [85].

Both extracts (*Trifolium* and *Ocimum*) are characterized by high content of antioxidants compounds, which are also responsible for the radiance and resistance of the skin and the slowing down of the aging process by maintaining estrogen levels [86]. Additionally, due to the existence of isoflavone-like compounds, the extracts of these plants quickly heal wounds and burns and reduce the chances of skin cancer [69,86].

These extracts can be used in multiple skin conditions such as acne, eczema boils, psoriasis and rashes because they help regenerate cells and have anti-inflammatory properties [59,87]. External applications are also beneficial to heal wounds, but they are less studied.

## 5. Therapeutic Activities and Mechanisms of Action for *Ocimum* sp. and *Trifolium* sp. Depending on the Type of Extraction Performed

The phytochemical profile of plant differs depending on the extraction method and solvents used to obtain their extracts [88]. Thus, organic solvents (ethanol, methanol) or hydroalcoholic mixtures are most commonly used for the extraction of phenol, flavonoid compounds [89]. To obtain volatile compounds steam distillation or cold pressing is most often used [90]. Thus, depending on the type and method of extraction, different compounds are extracted which will determine differentiated therapeutic effects [88]. The type of extract made on *Trifolium pratense* and *Ocimum basilicum*, respectively the therapeutic effects demonstrated in the specialized literature and their mechanism of action are presented in Table 1 and Table 2.

## 6. In Vitro Wound Healing Effect of the Mixture of *Trifolium pratense* and *Ocimum basilicum* Extracts

Due to the wound healing properties of both *Trifolium* and *Ocimum* species highlighted in the literature and mentioned in this paper, the future perspectives refer to the possible combinate effect of the two species extracts. The ability to promote wound healing by synergic effect of *Trifolium Pratense* and *Ocimum basilicum* mixt extract has not been studied yet, being the central point for future studies. So far, the anti-inflammatory, antimicrobial, antifungal and anticancer properties have been demonstrated for each extract individually, obtaining promising results, and for these reasons, in the future, the mixture of both extracts are of great interest to be studied, expecting for a potential synergistic effect.

It is known that antioxidant enzymes play a key role in wound healing [119,120], due to which we assume that the mixture of *Trifolium pretense* and *Ocimum basilicum* extracts would have an increased antioxidant potential leading to in vitro wound healing. Another aspect that led to future studies and applications of the extract mixture of *Trifolium pretense* and *Ocimum basilicum* is that in the wound healing process also intervenes the inflammatory phase, the mixture of both extracts having anti-inflammatory effect.

**Figure 4:**
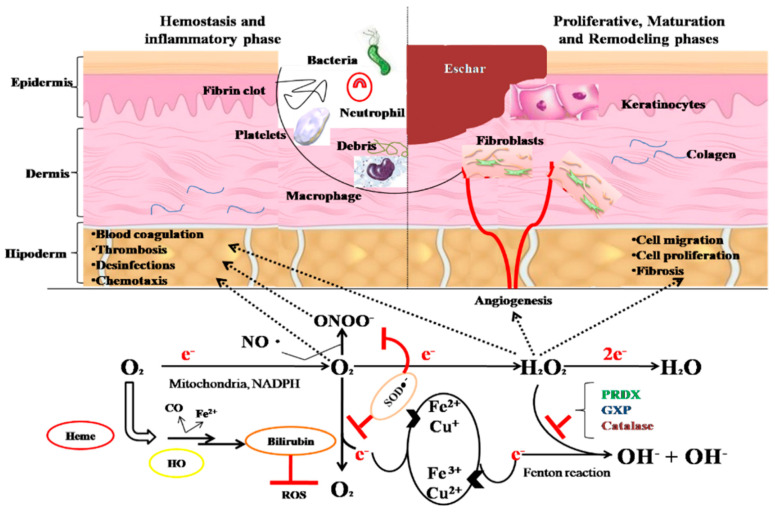
Stages of the wound healing process (adapted from [121]).

According to Figure 4, it is highlighted that the wound healing phases are reproduced through the processes of homeostasis, inflammation, blood coagulation with thrombus formation and natural disinfection of the wound. After these phases, healing stages are observed, represented by the migration and proliferation of dermal fibroblasts. All these steps are based on the biochemical reactions shown in Figure 4, which are strongly influenced and catalyzed by enzymes such as NADPH oxidase present in immune cells, superoxide dismutase (SOD) which catalyzes the reaction between superoxide and nitric oxide resulting in peroxynitrite (antibacterial) and the reaction of formation of hydrogen peroxide and molecular oxygen. Hydrogen peroxide formed by the reaction catalyzed by SOD is the key element that dictates the beginning of all stages of healing and re-epithelialization of the damaged area. Hydrogen peroxide must also be maintained at an optimal level to dictate the migration and proliferation of fibroblasts inside the wound and this level is maintained by a series of enzymes such as catalase, glutathione peroxidase (GXP) and peroxyredoxin (PRDX).

To the best of our knowledge, there are no studies in the literature dealing with any in vitro tests of the mixture *Trifolium pretense* and *Ocimum basilicum* extracts. Our research group performed a preliminary study using the “scratch test” assay on human fibroblasts, by applying the extract mixture in different concentrations on fibroblasts culture, in order to evaluate the optimum concentration to promote the stimulation and proliferation of the cells. Within this test, which is an in vitro model of wound healing, human fibroblasts were primarly grown to a confluent monolayer, and then was scraped in a straight line with a pipette tip, in order to simulate a wound. The fibroblasts migration into the wound area was monitored during 48 h incubation in the presence of different concentrations of mixed plant extracts along with the control (no treatment).

**Figure 5:**
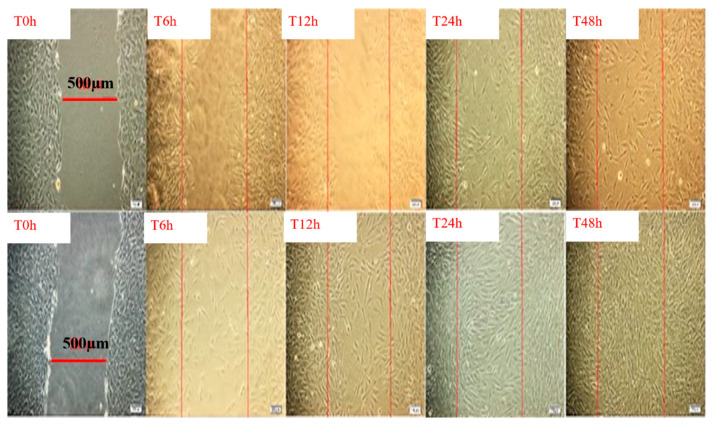
Migration of dermal fibroblasts after treatment with mixed extract of *Trifolium* pretense and *Ocimum basilicum* (lower line) compared to the control (upper line) monitored after different times intervals under light microscopy (objective 20×). Scale bar: 100 μm. The edge of initial pseudo-wound area is labeled in red, in order to emphasize the progressive covering of the area, during 48 h incubation (unpublished results).

**Figure 6:**
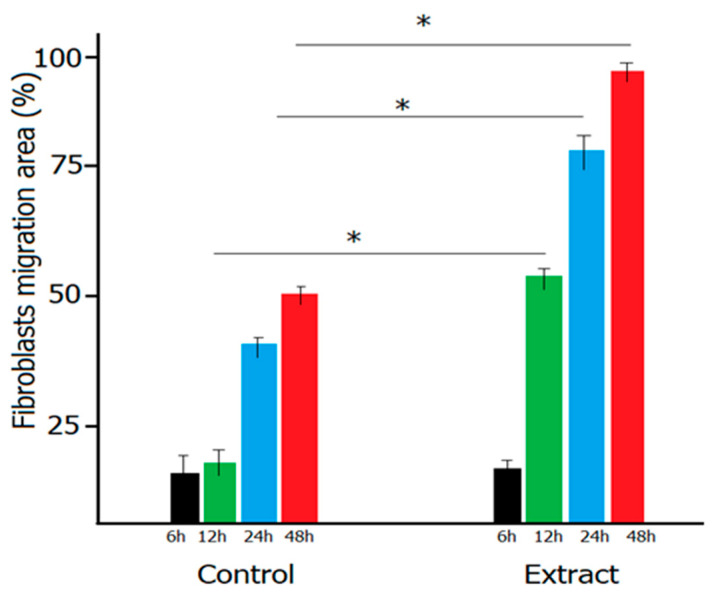
Wound healing percent express as fibroblast migration to cover the scratched area. Values are expressed as mean value of three independent measurements ± standard deviation. Statistically significant difference were considered for *p* < 0.05 (unpublished results).

In Figure 5, the spontaneous migration of dermal fibroblasts is evidenced under light microscopy, along with the control samples, showing the progressive covering of the pseudo-wound monitored at different times intervals. The percent of wound closure, expressed as migration of fibroblasts to cover the scratch area, is evidenced in Figure 6.

At the end of the monitoring period, a 100% coverage was achieved for the treated samples, compared to 52% for the control. These results are very promising, indicating that the mixture *Trifolium pretense* and *Ocimum basilicum* extract presents favorable biological activity to improve dermal regenerative process, being a good candidate to be used in both cosmetic and therapeutic formulations.

## 7. Conclusions

The antioxidant, antimicrobial, antiviral, antifungal and anti-inflammatory activity of *Ocimum* and *Trifolium* species are summarized in this review in order to explore the therapeutic potential of *Ocimum basilicum* and *Trifolium pretense* in relation with their phytochemical profile and to highlight the pharmacological activity of aqueous or ethanol extracts. Special attention was devoted to the dermal pathology and wound healing effects, in the context of multiple skin conditions such as acne, eczema boils, psoriasis and rashes. Both extracts (*Trifolium sp.* and *Ocimum sp.*) are characterized by high content of antioxidant compounds, which are also responsible for the radiance and resistance of the skin and the slowing down of the aging process by maintaining estrogen levels. Moreover, the potential combined effect of the mixed extract is pointed out in terms of future applications for wound healing, based on some preliminary results obtained from a “scratch tests” assay performed with respect to human dermal fibroblasts.

## Figures and Tables

**Table 1:** Biomedical activities of *Ocimum basilicum* in different extracts types.

Extract Type	Therapeutic Effect	Mechanism of Action	Ref.
Methanol extract	Wound healing effect	Angiogenesis stimulation (by cytokine activity modulation (TNF- α); Antimicrobial activity and antifungal activity; Antioxidative properties (Ferullic and Chlorogenic Acid).	[91,92,93,94]
	Hepatoprotection	Modulatory effect in hepatocytes comparable to oleanolic and ursolic acids	[67,74]
Ethanol extract	Dermatological effects	Strong antiviral activity against DNA viruses and RNA viruses; Effect on lipid accumulation in human macrophage	[95]
	Anti-cancer	Cytotoxic effect: increase of Glutathione S-Transferase (GST) activity and protection in carcinogenicity or toxicity (antioxidant activity, antiproliferative effect).	[96,97,98]
	Hypocholesterolemia	Lowering the lipid accumulation in human macrophage.	[99]
Hydroalcoholic extract	Adjuvant in diabetes treatment	Anti-hyperglycemic effect (antioxidant activity and inhibition of α-glucosidase and α-amylase activities).	[93]
	Vasorelaxant/anti-platelet effect	Anti-thrombotic effect (inhibits ADP and thrombin induced platelet aggregation)	[24,100,101,102]
	Neuro-psycho effects	Anxiolytic and sedative effect (action of malic, caffeic, kaempferol and oleanolic acids)	[101]
	Antiosteoporotic effect	Bone protection against osteoporosis induced by glucocorticoids.	[103]
	Anti-inflammatory effect	slight effect on Nitrogen Oxide synthesis, reduced leukocytes and monocytes, activation of phagocytes circulation	[104]
Essential oils	Treatment of different skin pathologies/antiaging	Enhancing the skin penetration in vitro animal experiments. Antioxidant capacity (major oil compounds: linalool, isoanethole, eugenol) comparable to tocopherol.	[105,106]
	Complementary with antibiotics	Synergic effect of Basil with some antibiotics for the treatment of certain bacterial infection (ex. Propionibacterium acne)	[107]
	Antitumoral effect	Cytotoxic activity (higher inhibition of the viability of Ehrlich ascites carcinoma cells due to linalool)	[108]
	Anti-Colitis treatment	Protective effect against colitis induced by acetic acid (significant decrease of myeloperoxidase).	[109]

**Table 2:** Biomedical activities of *Trifolium pratense* in different extracts types.

Extract Type	Therapeutic Effect	Mechanism of Action	Ref.
Methanolic extract	Wound healing effect	The genistein present in the extract stimulates angiogenesis by activating the beta estrogen receptor, both by mechanisms dependent on this receptor and by independent mechanisms regulating wound healing. Antioxidant (isoflavones: triterpene saponins and flavonoids). Anti-inflammatory: genistein achieved by the downregulation of proinflammatory mediator activity (inactivation of nuclear factor-κB (NF-κB) and reduction in the expression levels of TNF-α Antimicrobial Antifungal effect against: *Aspergillus niger*, *C. albicans* and *Fusarium verticillioides.*	[28,110,111,112,113,114]
	Antiaging	high concentration of phenolic compounds and flavonoids have the ability to reduce and neutralize free radicals in the skin	[115]
	Antiplatelet aggregation	activates the antiplatelet factor nitric oxide synthesis in the cells	[116]
Ethanolic extract	Antispasmodic	in laryngitis, whooping cough, bronchitis and tuberculosis causes relaxation of the smooth muscles of the airways with relief of spasms	[112]
Hydroalcoholic extract	Hepatoprotective	increases the level of methionine in hepatic steatosis	[100]
	Anti-diabetic	Ferulic acid inhibits the enzymes involved in the digestion of carbohydrates (α-amylase and α-glucosidase) and has anti-lipase activity.	[63,117]
	Anticancer	The dimeric alkaloids vinblastine and vincristine have anticancer properties due to their activity in destroying cancer cells. Polyphenolic compounds have a protective role and induce a reduction in the number of human tumor cells or an increase in them.	[57,118]

## Data Availability

Data available in a publicly accessible repository.

## Supplementary Materials

The following are available online at https://www.mdpi.com/article/10.3390/plants10071390/s1, Table S1: The chemical structures and therapeutic activities of the major compounds identified for *Ocicum sp.*, Table S2: The chemical structures and therapeutic activities of the major compounds identified for *Trifolium* sp.
