# Supplementary material for: Perspectives on the Combined Effects of Ocimum basilicum and Trifolium pratense Extracts in Terms of Phytochemical Profile and Pharmacological Effects
Source: Plants (Basel). 2021 Jul 7;10(7):1390. doi: 10.3390/plants10071390 (PMC8309466; doi:10.3390/plants10071390)
Supplement: Supplementary file 1 [file plants-10-01390-s001.zip › plants-1264649-supplementary.pdf]

Table S1. **The chemical structures and therapeutic activities of the major compounds identified for *Ocicum sp.***

	The name of the compound	3D Structure	Activity	Ref.
**Essential Oil**	Linalool	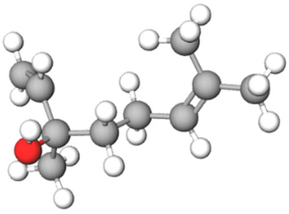	**Antioxidant, Anti-inflammatory, Antimicrobial Antiviral**	[1,2]
	1,8-Cineol	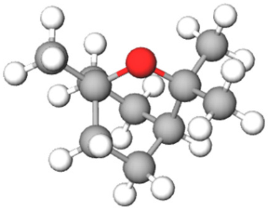	**Antioxidant, Anti-inflammatory, Antimicrobial**	[3,4]
	Eugenol	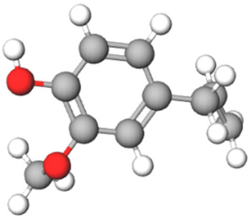	**Antioxidant, Repellent**	[2,5]
	Methyl-cinnamate	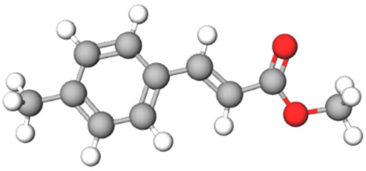	**Antimicrobial Analgesic**	[6]
	Carvacrol	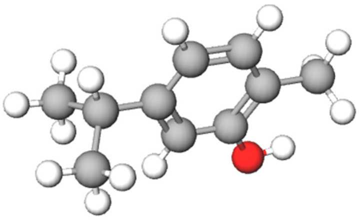	**Antibacterial, Anti-inflammatory, Inhibitory enzymatic activities**	[7,8]
**Hydroalcoholic extract**	Cinnamic acid	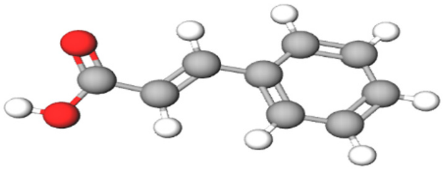	**Antimicrobial, Antioxidant, Anticancer,**	[9,10]

Caffeic acid	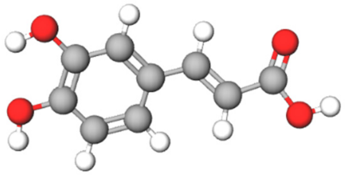	**Hepatoprotective, Nematicidal, Reduction of free radicals and oxidative stress**	
Ferulic acid	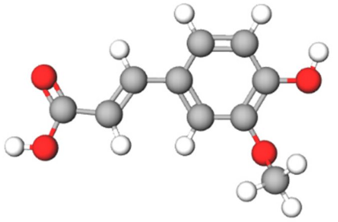		
Chlorogenic acid	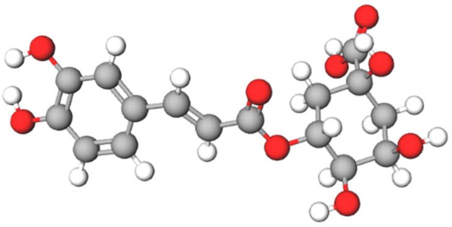		
Rosmarinic acid	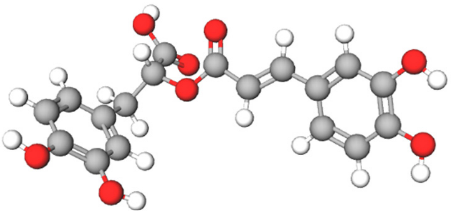		
Vanillic acid	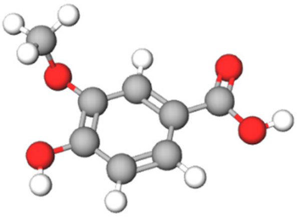		
Rutin	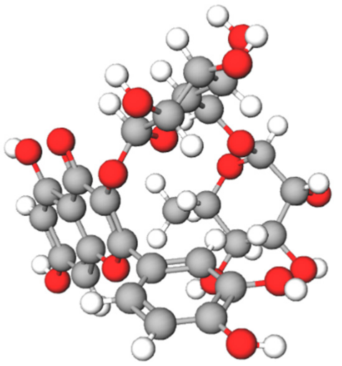	**Immunostimulant, Antioxidant, Antiviral,**	[11,12]

Apigenin	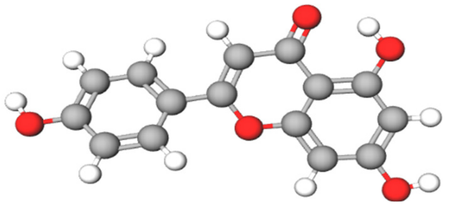	**Antidiabetic, Antioxidant, Anti-inflammatory, Anticancer, Wound healing, Antidepressant.**	[6,13]
Quercetin	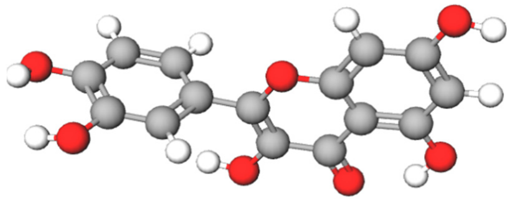		

Table S2. The chemical structures and therapeutic activities of the major compounds identified for *Trifolium sp.*

	**The name of the compound**	**3D Structure**	**Activity**	**Ref.**
**Hydroalcoholic extract**	Linamarin	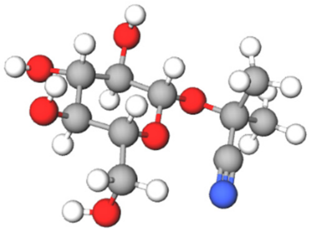	**Antitumoral, Anti-inflammatory, Antioxidant**	[1,14]
	Lotaustraline	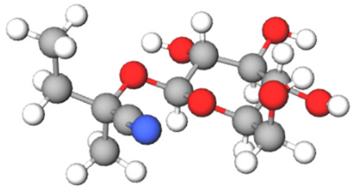		
	Formononetin	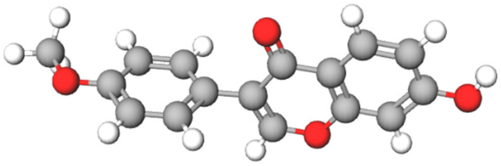	**Hepatoprotective, Antitumoral, Anti-inflammatory, Immunostimulatory.**	[15,16]

Daidzein	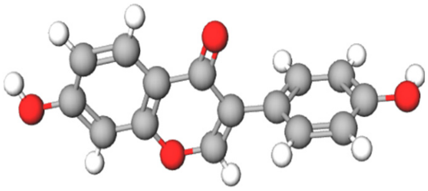	**Antioxidant Anticancer, Anti-inflammatory, Immunostimulatory, Estrogenic action.**	[14,17]
Genistein	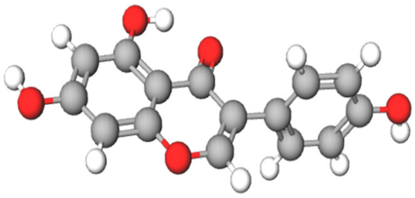		
Biochanin A	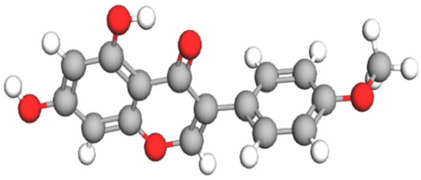	**Anticancer, Antiviral, Bone protections, Anti-inflammatory, Neuroprotective.**	[18,19]
Quercetin	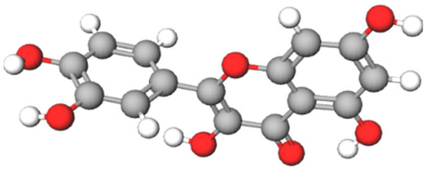	**Antidiabetic, Antioxidant, Anti-inflammatory, Anticancer, Wound healing, Antidepressant.**	[20]
Kaempferol	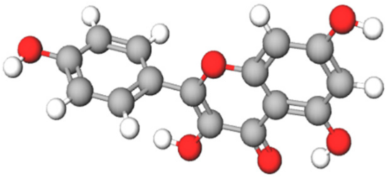		
Caffeic acid	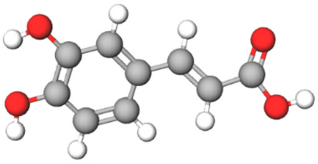	**Antimicrobial, Antioxidant, Anticancer, Hepatoprotective Nematicidal,**	[21]
Ferulic acid	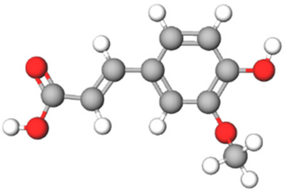		
Chlorogenic acid	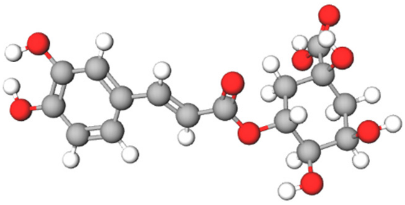		

Syringic acid	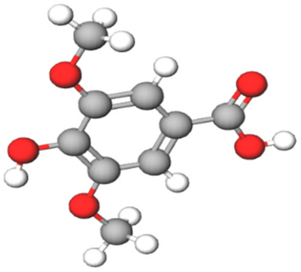	**Reduction of free radicals and oxidative stress, Antifungal.**	
Rutin	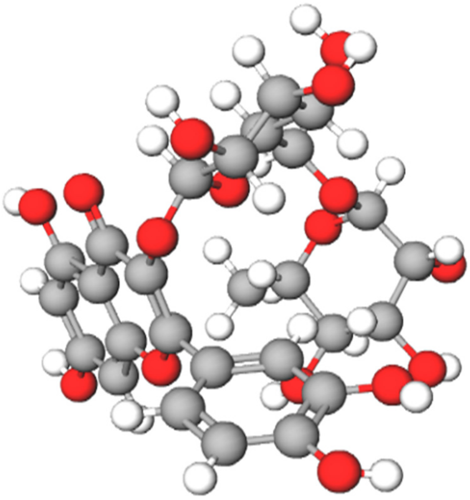	**Immunostimulant, Antioxidant, Antiviral.**	[22]

The 3D structures were made with the molview.org program. The carbon atoms in the structure are represented by gray, hydrogen is represented by white, nitrogen is represented by blue and oxygen atoms by red.

References

1. Chauhan, P. Skin Cancer and Role of Herbal Medicines. *Asian J Pharm Pharmacol* **2018**, *4*, 404–412, doi:10.31024/ajpp.2018.4.4.5.
2. Politeo, O.; Jukic, M.; Milos, M. Chemical Composition and Antioxidant Capacity of Free Volatile Aglycones from Basil (Ocimum Basilicum L.) Compared with Its Essential Oil. *Food Chemistry* **2007**, *101*, 379–385, doi:10.1016/j.foodchem.2006.01.045.
3. Araújo Couto, H.G.S. de; Blank, A.F.; Oliveira E Silva, A.M. de; Nogueira, P.C. de L.; Arrigoni-Blank, M. de F.; Nizio, D.A. de C.; Pinto, J.A. de O. Essential Oils of Basil Chemotypes: Major Compounds, Binary Mixtures, and Antioxidant Activity. *Food Chem* **2019**, *293*, 446–454, doi:10.1016/j.foodchem.2019.04.078.
4. Marian, E.; Vicas, L.G.; Jurca, T.; Muresan, M.; Pallag, A.; Stan, R.L.; Sevastre, B.; Diaconeasa, Z.; Ionescu, C.M.L.; Hangan, A.C. Salivia Officinalis L. and Verbascum Phlomoides L. Chemical, Antimicrobial, Antioxidant and Antitumor Investigations. *Revista de Chimie* **2018**, *69*, 365–370.
5. Fritea, L.; Pasca, P.M.; Vlase, L.; Gheldiu, A.-M.; Moldovan, L.; Banica, F.; Dobjanschi, L.; Cavalu, S. Electrochemical Methods for Evaluation of Antioxidant Properties of Propolis Extract Incorporated in Chitosan Nanoparticles. *Mater. Plast.* **2021**, *57*, 96–108, doi:10.37358/MP.20.4.5410.
6. Ali Khan, B.; Ullah, S.; Khan, M.K.; Alshahrani, S.M.; Braga, V.A. Formulation and Evaluation of Ocimum Basilicum-Based Emulgel for Wound Healing Using Animal Model. *Saudi Pharm J* **2020**, *28*, 1842–1850, doi:10.1016/j.jsps.2020.11.011.

7. Benedec, D.; Pârvu, A.E.; Oniga, I.; Toiu, A.; Tiperciuc, B. Effects of Ocimum Basilicum L. Extract on Experimental Acute Inflammation. *Rev Med Chir Soc Med Nat Iasi* **2007**, *111*, 1065–1069.
8. Diniz do Nascimento, L.; Moraes, A.A.B. de; Costa, K.S. da; Pereira Galúcio, J.M.; Taube, P.S.; Costa, C.M.L.; Neves Cruz, J.; de Aguiar Andrade, E.H.; Faria, L.J.G. de Bioactive Natural Compounds and Antioxidant Activity of Essential Oils from Spice Plants: New Findings and Potential Applications. *Biomolecules* **2020**, *10*, E988, doi:10.3390/biom10070988.
9. Flanigan, P.M.; Niemeyer, E.D. Effect of Cultivar on Phenolic Levels, Anthocyanin Composition, and Antioxidant Properties in Purple Basil (Ocimum Basilicum L.). *Food Chem* **2014**, *164*, 518–526, doi:10.1016/j.foodchem.2014.05.061.
10. Shahrajabian, M.H.; Sun, W.; Cheng, Q. Chemical Components and Pharmacological Benefits of Basil (Ocimum Basilicum): A Review. *International Journal of Food Properties* **2020**, *23*, 1961–1970, doi:10.1080/10942912.2020.1828456.
11. Saha, S.; Mukhopadhyay, M.K.; Ghosh, P.D.; Nath, D. Effect of Methanolic Leaf Extract of Ocimum Basilicum L. on Benzene-Induced Hematotoxicity in Mice. *Evidence-Based Complementary and Alternative Medicine* **2012**, *2012*, e176385, doi:10.1155/2012/176385.
12. Rezzoug, M.; Bakchiche, B.; Gherib, A.; Roberta, A.; FlaminiGuido, null; Kilinçarslan, Ö.; Mammadov, R.; Bardaweel, S.K. Chemical Composition and Bioactivity of Essential Oils and Ethanolic Extracts of Ocimum Basilicum L. and Thymus Algeriensis Boiss. & Reut. from the Algerian Saharan Atlas. *BMC Complement Altern Med* **2019**, *19*, 146, doi:10.1186/s12906-019-2556-y.
13. Salehi, B.; Venditti, A.; Sharifi-Rad, M.; Kręgiel, D.; Sharifi-Rad, J.; Durazzo, A.; Lucarini, M.; Santini, A.; Souto, E.B.; Novellino, E.; et al. The Therapeutic Potential of Apigenin. *International Journal of Molecular Sciences* **2019**, *20*, 1305, doi:10.3390/ijms20061305.
14. Yokoyama, S.-I.; Kodera, M.; Hirai, A.; Nakada, M.; Ueno, Y.; Osawa, T. Red Clover (Trifolium Pratense L.) Sprout Prevents Metabolic Syndrome. *J Nutr Sci Vitaminol (Tokyo)* **2020**, *66*, 48–53, doi:10.3177/jnsv.66.48.
15. Mediratta, P.K.; Sharma, K.K.; Singh, S. Evaluation of Immunomodulatory Potential of Ocimum Sanctum Seed Oil and Its Possible Mechanism of Action. *J Ethnopharmacol* **2002**, *80*, 15–20, doi:10.1016/s0378-8741(01)00373-7.
16. Al-Maskri, A.Y.; Hanif, M.A.; Al-Maskari, M.Y.; Abraham, A.S.; Al-sabahi, J.N.; Al-Mantheri, O. Essential Oil from Ocimum Basilicum (Omani Basil): A Desert Crop. *Nat Prod Commun* **2011**, *6*, 1487–1490.
17. Mukund, V.; Mukund, D.; Sharma, V.; Mannarapu, M.; Alam, A. Genistein: Its Role in Metabolic Diseases and Cancer. *Crit Rev Oncol Hematol* **2017**, *119*, 13–22, doi:10.1016/j.critrevonc.2017.09.004.
18. Zgonc Škulj, A.; Poljšak, N.; Kočevar Glavač, N.; Kreft, S. Herbal Preparations for the Treatment of Hair Loss. *Arch Dermatol Res* **2020**, *312*, 395–406, doi:10.1007/s00403-019-02003-x.
19. Yu, C.; Zhang, P.; Lou, L.; Wang, Y. Perspectives Regarding the Role of Biochanin A in Humans. *Front. Pharmacol.* **2019**, *10*, doi:10.3389/fphar.2019.00793.
20. Ahmad, S.; Zeb, A. Phytochemical Profile and Pharmacological Properties of Trifolium Repens. *J Basic Clin Physiol Pharmacol* **2020**, doi:10.1515/jbcpp-2020-0015.
21. Miere (Groza), F.; Teusdea, A.C.; Laslo, V.; Fritea, L.; Moldovan, L.; Costea, T.; Uivarosan, D.; Vicas, S.I.; Pallag, A. Natural Polymeric Beads for Encapsulation of Stellaria Media Extract with Antioxidant Properties. *Mat.Plast.* **2019**, *56*, 671–679, doi:10.37358/MP.19.4.5252.
22. Kanadys, W.; Baranska, A.; Jedrych, M.; Religioni, U.; Janiszewska, M. Effects of Red Clover (Trifolium Pratense) Isoflavones on the Lipid Profile of Perimenopausal and Postmenopausal Women-A Systematic Review and Meta-Analysis. *Maturitas* **2020**, *132*, 7–16, doi:10.1016/j.maturitas.2019.11.001.
